# *Galleria mellonella* as a Novelty *in vivo* Model of Host-Pathogen Interaction for *Malassezia furfur* CBS 1878 and *Malassezia pachydermatis* CBS 1879

**DOI:** 10.3389/fcimb.2020.00199

**Published:** 2020-05-05

**Authors:** Maritza Torres, Elkin Nicolás Pinzón, Flor Maria Rey, Heydys Martinez, Claudia Marcela Parra Giraldo, Adriana Marcela Celis Ramírez

**Affiliations:** ^1^Grupo de Investigación Celular y Molecular de Microorganismos Patógenos (CeMoP), Departamento de Ciencias Biológicas, Universidad de los Andes, Bogotá, Colombia; ^2^Unidad de Investigación en Proteómica y Micosis Humanas, Grupo de Enfermedades Infecciosas, Departamento de Microbiología, Facultad de Ciencias, Pontificia Universidad Javeriana, Bogotá, Colombia

**Keywords:** *Galleria mellonella*, *Malassezia furfur*, *Malassezia pachydermatis*, infection model, host-pathogen interaction

## Abstract

*Malassezia furfur* and *Malassezia pachydermatis* are lipophilic and lipid dependent yeasts, associated with the skin microbiota in humans and domestic animals, respectively. Although they are commensals, under specific conditions they become pathogens, causing skin conditions, such as pityriasis versicolor, dandruff/seborrheic dermatitis, folliculitis in humans, and dermatitis and otitis in dogs. Additionally, these species are associated with fungemia in immunocompromised patients and low-weight neonates in intensive care units with intravenous catheters or with parenteral nutrition and that are under-treatment of broad-spectrum antibiotics. The host-pathogen interaction mechanism in these yeasts is still unclear; for this reason, it is necessary to implement suitable new host systems, such as *Galleria mellonella*. This infection model has been widely used to assess virulence, host-pathogen interaction, and antimicrobial activity in bacteria and fungi. Some advantages of the *G. mellonella* model are: (1) the immune response has phagocytic cells and antimicrobial peptides that are similar to those in the innate immune response of human beings; (2) no ethical implications; (3) low cost; and (4) easy to handle and inoculate. This study aims to establish *G. mellonella* as an *in vivo* infection model for *M. furfur* and *M. pachydermatis*. To achieve this objective, first, *G. mellonella* larvae were first inoculated with different inoculum concentrations of these two *Malassezia* species, 1.5 × 10^6^ CFU/mL, 1.5 × 10^7^ CFU/mL, 1.5 × 10^8^ CFU/mL, and 11.5 × 10^9^ CFU/mL, and incubated at 33 and 37°C. Then, for 15 days, the mortality and melanization were evaluated daily. Finally, the characterization of hemocytes and fungal burden assessment were as carried out. It was found that at 33 and 37°C both *M. furfur* and *M. pachydermatis* successfully established a systemic infection in *G. mellonella*. *M. pachydermatis* proved to be slightly more virulent than *M. furfur* at a temperature of 37°C. The results suggest that larvae mortality and melanization is dependent on the specie of *Malassezia*, the inoculum concentration and the temperature. According to the findings, *G. mellonella* can be used as an *in vivo* model of infection to conduct easy and reliable approaches to boost our knowledge of the *Malassezia* genus.

## Introduction

The genus *Malassezia* is comprised of 18 lipophilic species characterized as commensal yeasts, which are part of the skin mycobiota of both humans and animals (Nagata et al., 2012; Cabañes, 2014; Cabañes et al., 2016; Honnavar et al., 2016; Prohic et al., 2016), and are commonly located in lipid-rich body areas, such as the scalp, face, and trunk (Gupta et al., 2004; DeAngelis et al., 2005). Some species of this genus have been associated with skin diseases (Gupta et al., 2004; Prohic et al., 2016) and systemic infections (Gaitanis et al., 2012; Kaneko et al., 2012; Nagata et al., 2012; Iatta et al., 2014, 2018; Roman et al., 2016; Lee et al., 2018; Theelen et al., 2018; Chen et al., 2020).

*Malassezia furfur* and *Malassezia pachydermatis* are two representative species of this genus which are found mainly in the skin of humans and pets such as dogs and cats, respectively (Cabañes, 2014). These two species have been associated with systemic infections in premature infants (Zomorodain et al., 2008; Iatta et al., 2014, 2018) as well as in low-weight infants with central venous catheters (CVC), parenteral lipid infusions and long hospital stays (Bell et al., 1988; Chang et al., 1998; Gupta et al., 2014), The two species have also been shown to infect immunosuppressed children and adults (Gaitanis et al., 2012; Kaneko et al., 2012; Roman et al., 2016); in fact, in some cases the percentage of fungemia by *M. furfur* (2.1%) is higher than fungemia by *Candida* spp. (1.4%) (Iatta et al., 2014), and prevalence can range from 2.1 to 4.4% in a neonatal intensive care unit depending on the diagnostic method (Iatta et al., 2018).

The skin colonization of neonates is the first step for the subsequent systemic invasion (Zomorodain et al., 2008; Iatta et al., 2014), a process that is not yet clear, and it has been reported that this may be facilitated by lipid infusion (Tragiannidis et al., 2010). This colonization has been reported from day zero to day 1 of life (Nagata et al., 2012), and it has been associated with transmission from the mother (Zomorodain et al., 2008; Nagata et al., 2012; Paul et al., 2019), healthcare personnel with pets (Chang et al., 1998), poor hygiene practices by healthcare personnel (handwashing before and after having contact with patients) leading to patient-patient transmission (Chang et al., 1998; Gaitanis et al., 2012), and persistence of *M. pachydermatis* yeasts in incubators and sheets (Iatta et al., 2014). Additionally, using fluconazole to prevent *Candida* spp. infections promotes colonization of resistant *M. furfur* (Chen et al., 2020).

Owing to the increase in the number of cases of *M. furfur* and *M. pachydermatis* systemic infections and the lack of knowledge of the infection mechanism, it is important to study the process of the systemic infection *in-vivo*. For this purpose, it is necessary to implement infection animal models. Different models have been used for the study of fungal diseases, such as zebrafish, chickens, mice, guinea pigs, dogs, and rabbits (McGinnis and Borgers, 1989; Capilla et al., 2007; Rosowski et al., 2018; Schlemmer et al., 2018; Sparber et al., 2019). These animal models have advantages such as the fact that they mimic human infectious diseases; however, their disadvantages are that they have high requirements for their maintenance, handling, space, and ethical considerations, which are the reasons as to why it is pivotal to implement alternatives (Capilla et al., 2007).

Among the alternatives are invertebrate organisms, such as *Drosophila melanogaster* used as a model for the study of *M. pachydermatis* infection (Merkel et al., 2018), *Caenorhabditis elegans* as an infection model of *Cryptococcus neoformans* (Capilla et al., 2007) and *Galleria mellonella*, which is widely used as a bacterial (Loh et al., 2013; Lacharme-Lora et al., 2019) and fungal infection model (Eisenman et al., 2014; Amorim-Vaz et al., 2015; Kloezen et al., 2015; Wuensch et al., 2018; Maurer et al., 2019; Sheehan and Kavanagh, 2019). One of the main features of *Galleria mellonella* that makes it a good model of mycosis are the phagocytic cells, which are the first line of defense against fungal infections (Trevijano-Contador and Zaragoza, 2019).

One of the advantages of using *G. mellonella* larvae as a model of host-pathogen interaction is the versatility of methods of inoculation, which can be topical (Scully and Bidochka, 2005), oral (Freitak et al., 2014) or the most common, microinjection (Eisenman et al., 2014; Amorim-Vaz et al., 2015; Kloezen et al., 2015; Wuensch et al., 2018; Sheehan and Kavanagh, 2019). Some other advantages include the feasibility of handling larvae due to their size, the fact that it is not necessary to feed them since they stop feeding in the fifth instar when experiments start (Singkum et al., 2019), the exclusion of ethical implications like those used in mammalian models, the ease of following-up on the progress of infection due to the use of standardized health scores (Champion et al., 2018), and the good antifungal immune response that includes cellular and humoral components (Kay et al., 2019; Trevijano-Contador and Zaragoza, 2019).

Due to the aforementioned factors, *G. mellonella* is an attractive *in-vivo* model of host-pathogen interaction that has been widely used in the evaluation of the host-pathogen interaction of yeasts such as *Candida* and *Cryptococcus*, but there are no reports, so far, on the use of *G. mellonella* as an infection model for *M. furfur* or *M. pachydermatis*. Therefore, the objective of this study is to establish *G. mellonella* as an infection model for *M. furfur* and *M. pachydermatis*, which will allow other mycologists to use this model in future studies. In this work, the survival of *G. mellonella* inoculated with different inoculum concentrations of *M. furfur* and *M. pachydermatis* was evaluated, and also the progress of the infection was followed by monitoring the melanization of the larvae and histological samples.

## Materials and Methods

### Fungal Strains and Inoculum Preparation

Strains of *M. furfur* CBS 1878 and *M. pachydermatis* CBS 1879 (Westerdijk institute, Utrecht, The Netherlands) were used. The strains were cultured for 72 h in the modified Dixon (mDixon) agar (36 g.L^−1^ mycosel agar [BD, USA], 20 g L^−1^ Oxgall [BD, USA], 36 g L^−1^ malt extract [Oxoid, UK], 2 mL L^−1^ glycerol [Sigma Aldrich, USA], 2 mL L^−1^ oleic acid [Sigma Aldrich, USA], and 10 mL L^−1^ Tween 40 [Sigma Aldrich, USA]) at 33°C. The inoculum was prepared with 5 mL of 0.5% Tween 80 [Sigma Aldrich, USA]. Then it was filtered and cells were counted in a Neubauer chamber to reach desirable inoculum concentrations: 1.5 × 10^6^ CFU/mL, 1.5 × 10^7^ CFU/mL, 1.5 × 10^8^ CFU/mL, and 1.5 × 10^9^ CFU/mL.

### Survival and Melanization Assay

**Figure 1:**
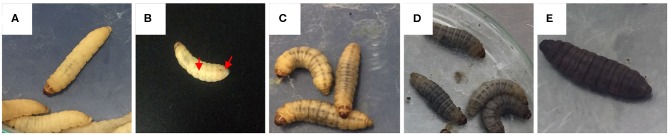
Melanization scores based on the health index scoring system. **(A)** melanization score 4, no melanization; **(B)** melanization score 3, <3 spots on beige larva (black arrows showing the spots); **(C)** melanization score 2, more than three spots on beige larva; **(D)** melanization score 1, black spots on brown larva; and **(E)** melanization score 0, full melanization.

Killing assays were performed in *G. mellonella* as previously described (Stephens, 1962; Fallon et al., 2012). Briefly, the larvae in late stages (fifth and sixth instar) were obtained from Perkins LTDA (Palmira, Colombia). Once the larvae arrived, they were kept at room temperature for 24 h, in that order, they could recover until challenge assay; then, in continuous movement, the larvae were decontaminated in 0.1% sodium hypochlorite for 30 s and rinsed in two different containers with sterile distilled water, in the first recipient for 30 s and the second recipient for 10 s. Larvae with a weight between 250 and 330 mg and a size of ~2 cm were selected and sorted in five different treatment groups of fifteen larvae, avoiding those with any kind of mark. Insulin syringes (BD Veo insulin syringe with the BD Ultra-Fine 6 mm needle) were used to microinject the larvae, holding them between the fingers, allowing the needle to insert at the last left proleg to deliver the inoculum directly into the hemocoel. Fifteen larvae for each treatment were inoculated with 20 μL of 0.5% Tween 80 as a negative control, and four inoculum concentrations (range 1.5 × 10^6^-1.5 × 10^9^ CFU/mL) Additionally, fifteen larvae were used as absolute control; these larvae were kept in the same conditions as the other five groups, but without any intervention to evaluate each lot of larvae that was received. After that, larvae were incubated at 33 and 37°C in darkness. Every 24 h during a 15 days period, larval silk was removed and the insects' death and melanization were recorded (Amorim-Vaz et al., 2015; Maurer et al., 2019). Larvae were scored as dead when they did not respond to a mechanical stimulus (Eisenman et al., 2014; Cools et al., 2019). Melanization was scored according to a modified health index scoring system (Figure 1) (Loh et al., 2013; Tsai et al., 2016; Champion et al., 2018; Kay et al., 2019). To compare mortality, three biological replicates were performed.

### Characterization of Hemocytes

To characterize hemocytes, two larvae from each treatment were collected at random, then a puncture in the last segment of the insect was made, allowing a drop of hemolymph to drain over a glass slide. The hemolymph drop was then spread over and stained with Wright's stain. According to previous reports (Arteaga Blanco et al., 2017; Wojda, 2017; Boguś et al., 2018), hemocytes were characterized and counted under a light Leica DM500 microscope.

### Fungal Burden

To access the fungal burden, three larvae per treatment were randomly collected. Their surfaces were cleaned by submerging the larvae in 0.1% sodium hypochlorite for 5 min and rinsing them twice in sterile distilled water for 1 min each time. Then, each larva was placed in 2 mL Eppendorf tubes with 1 mL of 0.5% Tween 80 each and ground for 5 min (Mylonakis et al., 2005). 100 μL of these solutions were used to make two serial dilutions, which were plated on mDixon agar at 33°C. The numbers of colony-forming units (CFU) were counted daily until no new colonies were observed.

### Histopathological Analysis

On day 5, two random larvae incubated at 37°C were fixed for 10 days at 4°C in 4% paraformaldehyde in PBS (4% PFA/BS), also these larvae were injected in the left proleg with 10 μL of 4% PFA/PBS (Perdoni et al., 2014; Kloezen et al., 2015; Wuensch et al., 2018). After 10 days, they were transferred to 70% ethanol for storage (Eisenman et al., 2014; Maurer et al., 2019). Before the paraffin protocol, larvae were dissected and observed under a Leica Zoom ^TM^2000 stereomicroscope; then, these larvae were embedded in paraffin, cut in sections of 5 μm and stained with hematoxylin-eosin. Nodules of larvae infected with *M. furfur* and *M. pachydermatis* inoculum concentration of 1.5 × 10^9^ CFU/mL were dissected apart and stained with calcofluor white [Sigma Aldrich, USA] and 2 mL of 10% KOH [Sigma Aldrich, USA] as described elsewhere (Sheehan and Kavanagh, 2019). Then, the nodules were observed under an Olympus FV1000 laser scanning confocal microscope at the microscopy core, μ-core, at the Universidad de los Andes.

### Statistical Analysis

All experiments were performed on three independent biological replicates; survival curves were constructed using the method of Kaplan and Meier, then the curves were compared using the Log-Rank (Mantel-Cox) test. Fungal burden, melanization score, and hemocyte count analysis were conducted using the 2-way ANOVA test. Statistical models were constructed and analyzed using the GraphPad Prism 8 software (version 8.2.0). A *p*-value under 0.05 was considered to be statistically significant.

## Results

### Survival and Melanization Assay

**Figure 2:**
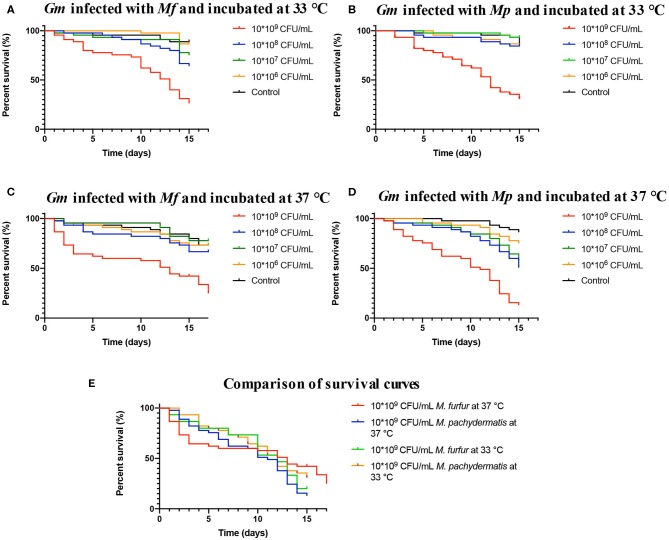
Survival curves of *G. mellonella* infected with *M. furfur* and *M. pachydermatis*. **(A)** The survival curve of *G. mellonella* infected with *M. furfur* and incubated at 33°C, 50% of mortality is reached on day 13. **(B)** The survival curve of *G. mellonella* infected with *M. pachydermatis* and incubated at 33°C, 50% of mortality is reached at day 12. **(C)** The survival curve of *G. mellonella* infected with *M. furfur* and incubated at 37°C, 50% of mortality is reached at day 13. **(D)** The survival curve of *G. mellonella* infected with *M. pachydermatis* and incubated at 37°C, 50% of mortality is reached at day 12. In general, larvae infected with an inoculum of 1.5 × 10^9^ CFU/mL had higher mortality (*p* < 0.0001, log-rank (Mantel-Cox) test). **(E)** Comparison of survival curves of larvae infected with inoculum of 1.5 × 10^9^ CFU/mL of *M. furfur* and *M. pachydermatis* incubated at 33 and 37°C, no significant differences were found between the four treatments (*p* = 0.2046, log-rank (Mantel-Cox) test). In all cases, survival depended on inoculum concentration. *Mf, M. furfur; Mp, M. Pachydermatis; Gm, G. mellonella*.

To determine the virulence of *M. furfur* and *M. pachydermatis* at 33 and 37°C, larvae were inoculated with four different inoculum concentrations. The first temperature (33°C) was used to identify whether *M. furfur* and M *pachydermatis* could establish a systemic infection in *G. mellonella*. At 33°C, it was found that *G. mellonella* larvae showed a differential survival behavior depending on the inoculum concentration (Figures 2A,B). Larvae inoculated with an inoculum concentration of 1.5 × 10^9^ CFU/mL of the two species of *Malassezia* showed significantly decreased survival (*p* < 0.0001, log-rank [Mantel-Cox] test). In the case of larvae infected with *M. furfur*, 50% of mortality was reached on day 13 (Figure 2A), while larvae infected with *M. pachydermatis* reached 50% of mortality on day 12 (Figure 2B).

Survival of control larvae and larvae infected with the remaining three concentrations of *M. furfur* and *M. pachydermatis* (1.5 × 10^6^, 1.5 × 10^7^, and 1.5 × 10^8^ CFU/mL) exhibited different patterns; larvae infected with *M. furfur* showed that at a lower concentration the larval survival increases, but it is still lower compared to the control larval survival (*p* = 0.014, log-rank [(Mantel-Cox]) test). In the case of larvae infected with *M. pachydermatis*, no significant difference in larval survival was found between control larvae and inoculum concentration of 1.5 × 10^6^ CFU/mL, and for the two remaining inoculum concentrations (1.5 × 10^7^ and 1.5 × 10^8^ CFU/mL), it was observed that larval survival decreased (*p* < 0.0001, log-rank [(Mantel-Cox]) test). After, it was demonstrated that *M. furfur* and *M. pachydermatis* were able to establish a systemic infection in *G. mellonella* larvae at 33°C, experiments were conducted at 37°C to evaluate the systemic infection at the human corporal temperature.

Larvae infection assays conducted at 37°C showed a decrease in survival (Figures 2C,D). Since day one, dead larvae were found in challenged larvae groups infected with different inoculum concentrations of *M. furfur* and *M. pachydermatis*. A significant decrease in larval survival was observed in larvae infected with *M. furfur* inoculum concentration of 1.5 × 10^9^ CFU/mL (*p* < 0.0001, log-rank (Mantel-Cox) test), reaching 50% of mortality on day 13, and larval death was observed since day 1. However, no larvae were reported dead from day 6 until day 10 (Figure 2C). In the case of larvae infected with *M. pachydermatis*, a significant difference in survival was found (*p* < 0.0001, log-rank [Mantel-Cox] test); larvae inoculated with the highest inoculum concentration (1.5 × 10^9^ CFU/mL) reached 50% of larval mortality on day 11, and larval death paused from 7 to day 10 (Figure 2D).

Control larvae and larvae inoculated with *M. furfur* inoculum concentrations of 1.5 × 10^6^, 1.5 × 10^7^, and 1.5 × 10^8^ CFU/mL did not show a significant difference in survival (*p* = 0.5439, log-rank [(Mantel-Cox]) test). On the other hand, larvae inoculated with *M. pachydermatis* showed a difference in survival when comparing the larval survival of those infected with the three remaining inoculum concentrations and the control larvae (*p* = 0.0010, log-rank [(Mantel-Cox]) test). A higher survival in control larvae and larvae inoculated with inoculum concentration of 1.5 × 10^6^ CFU/mL than in larvae with inoculum concentrations of 1.5 × 10^7^ and 1.5 × 10^8^ CFU/mL was found. The resulting data shows that survival at 37°C also depends on inoculum concentration. Survival curves of larvae inoculated with *M. furfur* and *M. pachydermatis* inoculum concentration of 1.5 × 10^9^ CFU/mL and incubated at 33 and 37°C were compared (Figure 2E), and no significant difference was found (*p* = 0.2046, log-rank [(Mantel-Cox]) test).

**Figure 3:**
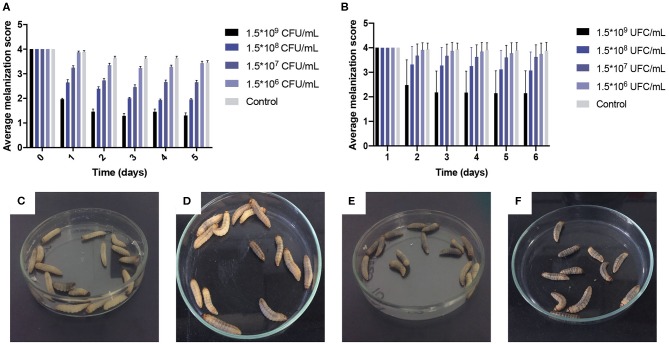
Average melanization scores of larvae infected with *M. furfur* and *M. pachydermatis* at 37°C and melanization comparison between *G. mellonella* infected with *M. furfur* at 33 and 37°C on day 7. As can be seen, melanization score depends on time and treatment; scores decreased as inoculum concentrations increase (*p* < 0.0001, 2-way ANOVA test). **(A)** Larvae infected with *M. furfur*. **(B)** Larvae infected with *M. pachydermatis*, moreover in **(C)** *G. mellonella* infected with 1.5 × 10^8^ CFU/mL inoculum concentration of *M. furfur* and incubated at 37°C, melanization was scored as 2 with beige larvae; but in **(D)** *G. mellonella* infected with 1.5 × 10^8^ CFU/mL inoculum concentration of *M. furfur* and incubated at 33°C, melanization score was recorded as two with darker larvae. Finally, in **(E)** *G. mellonella* infected with 1.5 × 10^9^ CFU/mL inoculum concentration of *M. furfur* and incubated at 37°C showed larvae with lower melanization than **(F)** *G. mellonella* larvae infected with 1.5 × 10^9^ CFU/mL inoculum concentration of *M. furfur* and incubated at 33°C. Larval melanization is higher in larvae incubated at 33°C.

The development of nodules with deposition of melanin is one of the main components of the immune response of *G. mellonella*. These nodules are visible macroscopically as brown spots, and in order to follow the progression of infection, the development of melanin nodules was monitored at 37°C. According to the scoring system used in this study, the melanization depends on time and the inoculum concentration of *M. furfur* and *M. pachydermatis* (Figures 3A,B). Melanization in larvae infected with both species of *Malassezia* showed to increase over time, with a significant decrease in the melanization score (*p* < 0.0001, 2-way ANOVA test). This melanization score showed a significant difference between control larvae and inoculated larvae (*p* < 0.0001, 2-way ANOVA test). The development of brown spots increased proportionally with the increase of inoculum concentration. The lowest melanization score was recorded in larvae inoculated with the highest concentration of both species of *Malassezia* (the melanization score range from 2 to 1). Finally, for *M. furfur*, it was observed that larvae incubated at 33°C exhibited higher melanization than those incubated at 37°C (Figures 3C–F).

### Hemocytes Characterization

**Figure 4:**
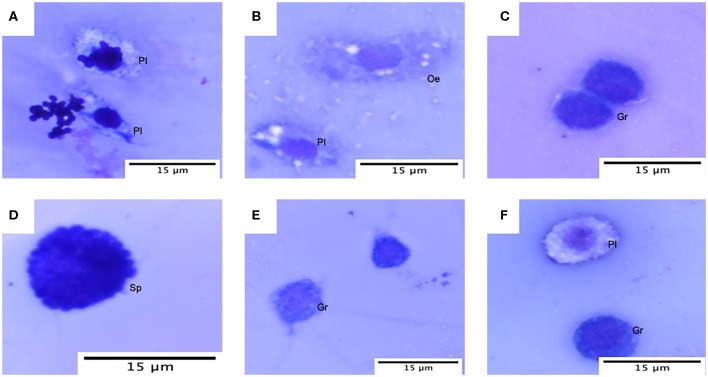
Hemocytes of *G. mellonella* under the light microscope. **(A)** Plasmatocytes (Pl) with *M. furfur* yeast; **(B)** oenocyte (Oe) and plasmatocyte (PI); **(C)** granulocyte (Gr); **(D)** spherulocyte (Sp); **(E)** granulocyte and prohemocyte (Pr); and **(F)** plasmatocyte and granulocyte.

**Figure 5:**
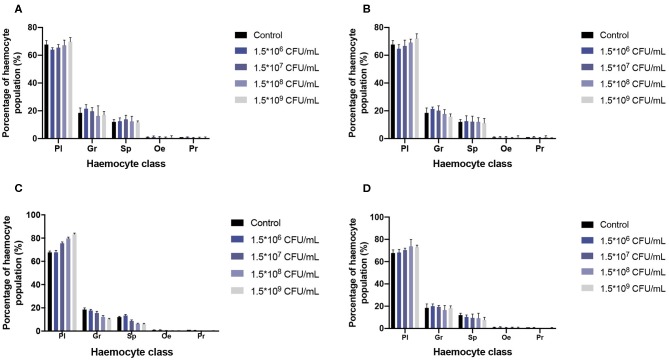
Percentage of hemocyte population. A significant difference was found in hemocyte populations when the interaction between hemocyte class and inoculum concentrations was analyzed (*p* < 0.05, 2-way ANOVA test). As can be noted, plasmatocytes tend to increase as the inoculum concentracion increases; on the other hand, Granulocytes, spherulocytes, oenocytes, and prohemocytes tend to decrease as the inoculum concentracion increases. **(A)** Percentage of hemocytes population of *G. mellonella* larvae infected with *M. furfur* at 33°C; **(B)** percentage of hemocytes population of *G. mellonella* larvae infected with *M. pachydermatis* at 33°C; **(C)** percentage of hemocytes population of *G. mellonella* larvae infected with *M. furfur* at 37°C; and **(D)** percentage of hemocyte population of *G. mellonella* larvae infected with *M. pachydermatis* at 37°C. Pl, plasmatocytes; Gr, granulocyte; Sp, spherulocyte; Oe, oenocyte; and Pr, prohemocyte.

Hemocyte population percentages were obtained and tabulated to characterize the immune response of *G. mellonella* and determine whether or not the infection of the two species of *Malassezia* has an effect on hemocyte populations. Cell classification was carried out based on size and morphology (Figure 4) (Arteaga Blanco et al., 2017; Wojda, 2017; Boguś et al., 2018). In both cases of *M. furfur* and *M. pachydermatis* infection assays at 33 or 37°C, a significant difference was found between cell populations (*p* < 0.0001, 2-way ANOVA test). No significant differences were found between any of the five treatments at 33 and 37°C (for larvae infected with *M. furfur* incubated at 33°C, *p* = 0.8076; at 37°C *p* = 0.9582 and for larvae infected with *M. pachydermatis* at 33°C, *p* = 0.9999; at 37°C, *p* = 0.9907, 2-way ANOVA test). However, the interaction between type of hemocytes and the treatment showed a significant difference in the percentage of hemocyte populations for larvae infected with *M. furfur* and *M. pachydermatis* at any of the two temperatures (*p* < 0.05, 2-way ANOVA test). The pattern observed indicates that the percentage of plasmocytes tends to increase, and the percentages of granulocytes, spherulocytes, oenocytoids, and prohemocytes decrease as the inoculum concentration increases for both larvae infected with *M. furfur* or *M. pachydermatis* (Figure 5).

### Fungal Burden

**Figure 6:**
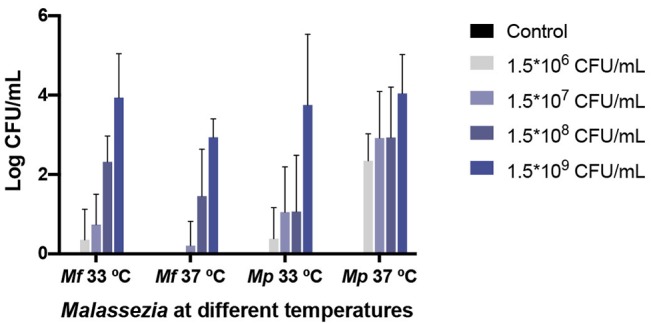
*M. furfur* and *M. pachydermatis* isolated from challenged *G. mellonella* larvae. No significant difference was found in yeast isolation of the two *Malassezia* species (*p* < 0.4068, 2-way ANOVA test). However, the recovery of yeasts from larvae challenged with *M. furfur* differed depending on temperature. At 33°C the fungal burden decreased but, in less proportion, than at 37°C. On the contrary, *M. pachydermatis* isolated from *G. mellonella* showed a better recovery at 33°C, similar yeast recovery was obtained for the highest inoculum concentration used. In all cases, the fungal burden demonstrated a decrease from the original inoculum concentration. *Mf, M. furfur; Mp, M. pachydermatis*.

No significant difference in yeast isolation was found between any treatment (larvae infected with *M. furfur* or *M. pachydermatis* and incubated at 33 and 37°C) (*p* < 0.4068, 2-way ANOVA test). However, as can be seen in Figure 6, *M. furfur* isolation from larvae incubated at 37°C was lower than from those incubated at 33°C. The yeast isolation from larvae inoculated with 1.5 × 10^9^ CFU/mL and incubated at 37°C was 10-folds lower than from those incubated at 33°C; as matter of fact, there was no isolation of yeasts from larvae inoculated with the lowest inoculum concentration and incubated at 37°C.

On the contrary, *M. pachydermatis* was isolated in higher proportion from larvae incubated at 37°C than from larvae incubated at 33°C, with the isolation of yeast from larvae inoculated with 1.5 × 10^9^ CFU/mL and incubated at 37°C, 2-folds higher than from larvae incubated at 33°C. At both temperatures, the *M. pachydermatis* burden was higher than the *M. furfur* burden in *G. mellonella* larvae, with the exception of larvae inoculated with the highest concentration and incubated at 33°C, in which the yeast isolation was 1.5-folds higher from larvae inoculated with *M. furfur* than from larvae inoculated with *M. pachydermatis*. At 37°C and using the highest inoculum concentration, the yeast recovery was 12-folds higher from larvae inoculated with *M. pachydermatis* than from larvae inoculated with *M. furfur*. In all cases, the fungal burden decreased compared to the original inoculum concentrations and it depended on its original inoculum concentration.

### Histopathological Analysis

**Figure 7:**
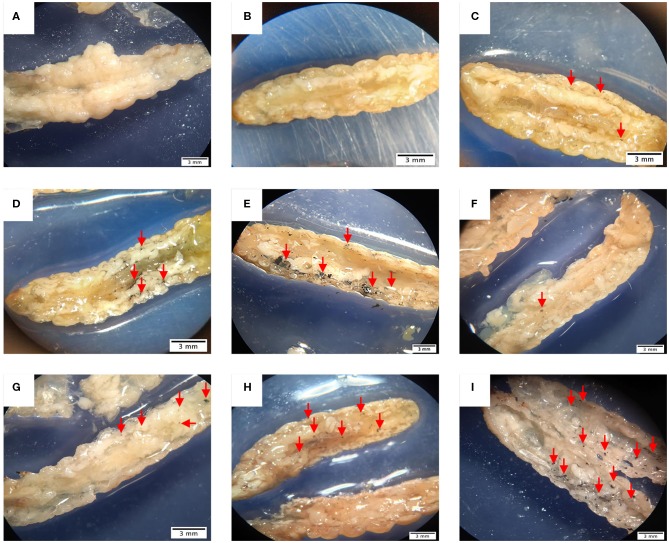
*G. mellonella* larvae half sectioned under the stereoscope. **(A)** Control larva showing no presence of nodules, in contrast **(B–E)** larvae infected with *M. furfur* displaying nodule formation close to the cuticle and lesser dissemination than **(F–I)** larvae infected with *M. pachydermatis*. In both cases, larvae present different quantity of nodules depending on the inoculum concentration, as the concentration increases, the number of nodules arise. Larvae infected with *M. furfur* **(B)** 1.5 × 10^6^ CFU/mL inoculum concentration, **(C)** 1.5 × 10^7^ CFU/mL inoculum concentration, **(D)** 1.5 × 10^8^ CFU/mL inoculum concentration, and **(E)** 1.5 × 10^9^ CFU/mL inoculum concentration. Larvae infected with *M. pachydermatis* **(F)** 1.5 × 10^6^ CFU/mL inoculum concentration, **(G)** 1.5 × 10^7^ CFU/mL inoculum concentration, **(H)** 1.5 × 10^8^ CFU/mL inoculum concentration, and **(I)** 1.5 × 10^9^ CFU/mL inoculum concentration. Red arrows show the position of nodules.

In order to evaluate the dissemination of *M. furfur* and *M. pachydermatis* at 37°C, larvae were sectioned into two halves and observed. As can be seen in Figure 7, larvae infected with either of the two species of *Malassezia* showed an increase in nodule occurrence as the inoculum concentration increased. In the case of larvae infected with *M. furfur*, it was perceived that the occurrence of nodules was concentrated to the periphery near the site of microinjection; in fact, this was easier to see in larvae infected with the highest concentration (Figures 7A–E). On the other hand, larvae infected with *M. pachydermatis* exhibited higher dissemination and occurrence of small nodules through the hemocoel (Figures 7F–I).

**Figure 8:**
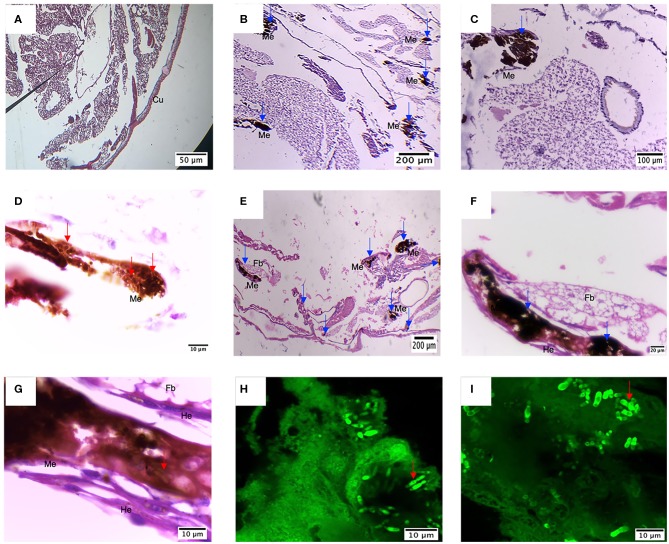
Histology of *G. mellonella* infected with *M. furfur* and *M. pachydermatis*. **(A)** Control larvae display high tissue integrity and no sign of nodule formation, different from this **(B–D)** larvae infected with *M. furfur* presented different size nodules with melanin deposition and recruitment of new hemocytes; this is similar to what happens to **(E–G)** larvae infected with *M. pachydermatis*, which present different nodule formation with hemocytes recruited around it and proximity to fat bodies. Red arrows point to the presence *of M. furfur* and *M pachydermatis* yeasts; blue arrows indicate the presence of nodules. Dissected nodules stained with Calcofluor white confirm infection by **(H)** *M. furfur* and **(I)** *M. pachydermatis*. Fb, fat bodies; He, hemocytes; Me, Melanin deposition.

Once the presence of the nodules and the pattern of dissemination was observed, larvae were embedded in paraffin and sectioned to identify changes in larval tissue and the immune response, as well as to confirm the establishment of the infection. First, comparing infected larvae with control larvae, the loss of tissue integrity is evident (Figure 8). Besides, larvae infected with *M. furfur* inoculum concentration of 1.5 × 10^9^ CFU/mL showed the presence of several nodules with melanin deposition of different sizes (Figure 8B). An enlarged image of one of the nodules (100X) shows the presence of newly recruited hemocyte around it (Figure 8C). Finally, yeasts of *M. furfur* are shown in the magnified nodule (1000X) (Figure 8D).

Compared to *M. furfur*, larvae infected with *M. pachydermatis* also exhibited nodule formations and higher hemocyte recruitment (Figures 8E,F). Inside the nodule, it is possible to observe yeasts of *M. pachydermatis* (Figure 8G); in both cases, the structures were found close to the fat bodies. To confirm the infection by either of the species of *Malassezia*, yeasts were stained with calcofluor white and observed under the confocal microscope. In contrast to *M. furfur*, a higher number of *M. pachydermatis* was found inside the nodules (Figures 8H,I).

## Discussion

*G. mellonella* has been successfully implemented as an infection model of fungal pathogens, like *Madurella mycetomatis* (Kloezen et al., 2015), *Cryptococcus neoformans* (Eisenman et al., 2014), *Aspergillus flavus* (Scully and Bidochka, 2005), *Candida albicans* (Amorim-Vaz et al., 2015), *Candida tropicalis* (Mesa-Arango et al., 2013), and dermatophytes (Achterman et al., 2011; Ishii et al., 2017). This model allows the evaluation of virulence factors, anti-fungal agents and immune response to a pathogen. Compared to other animal models, *G. mellonella* larvae are easy to handle, cheap, have no ethical limitations, and their innate immune system is similar to that of humans (Fuchs et al., 2010; Pereira et al., 2018; Singkum et al., 2019; Trevijano-Contador and Zaragoza, 2019). Besides, as shown in this study, larvae can be easily roomed at the desired temperature in an incubator, allowing the researchers to have better control of the experimental condition and to get reliable and reproducible data.

In this study, the establishment of the systemic infection of *M. furfur* and *M. pachydermatis* in *G. mellonella* was tested, two species belonging to the Genus *Malassezia* that have been related to opportunistic infection in the bloodstream in newborns and immunocompromised patients (Dokos et al., 2011; Gaitanis et al., 2012; Al-Sweih et al., 2014; Iatta et al., 2014; Chen et al., 2020). For the first time, it has been demonstrated that *M. furfur* and *M. pachydermatis* can establish a systemic infection in *G. mellonella* larvae. Also, it was shown that the larval survival depends on inoculum concentration; at the highest inoculum concentration tested (1.5 × 10^9^ CFU/mL), 50% of mortality was reached between day 12 and 13 for both species.

Furthermore, it was found that *M. furfur* at low concentrations can affect larval survival when they are incubated at 33°C, contrary to larvae incubated at 37°C in which larval survival is only affected at the highest inoculum concentration (1.5 × 10^9^ CFU/mL). In contrast, larvae inoculated with *M. pachydermatis* showed a decrease in larval survival at any temperature, even in low concentrations. Larval survival variations at different temperatures for *M. furfur* and *M. pachydermatis* infection could be related to their ecology, since *M. pachydermatis* is mainly found in domestic animals, like dogs and cats, which mean temperatures are 38.2 and 38.1°C, respectively (Kabatchnick et al., 2016). On the other hand, *M. furfur* is primarily found in human beings with a lower corporal temperature than the mentioned animals, leading to the belief that *M. pachydermatis* is better adapted to a higher temperature than *M. furfur*. Indeed, it has been reported in previous studies that *M. pachydermatis* has its optimal growing temperature between 32 and 37°C (Bond and Lloyd, 1996); on the other hand, the optimal growing temperature of *M. furfur* is around 34°C (Leeming and Notman, 1987).

Phospholipase activity is an important virulence factor in tissue invasion in chronic otitis in animals; in fact, strains of *M. pachydermatis* isolated from dogs with otitis showed higher expression and activity of phospholipases than strains isolated from healthy animals (Cafarchia and Otranto, 2004; Ortiz et al., 2013). Also, it was shown that *M. pachydermatis* has a high lipase and phospholipase activity in comparison to *M. furfur* (Juntachai et al., 2009). Herein, *M. pachydermatis* showed to decrease larval survival at any concentration and at both temperatures. In contrast, *M. furfur* showed a decrease in the negative effect on larval survival at 37°C and the negative effect on larval survival at 33°C was lower than the negative effect of *M. pachydermatis*. This may be a consequence of the phospholipase activity of *M. pachydermatis* that allows invasion causing damage to the host, making it more pathogenic than *M. furfur*.

Larval mortality paused from day 6 to 10 for larvae infected with either *M. furfur* or *M. pachydermatis* and incubated at 37°C. This may be due to the fact that hemocytes in *G. mellonella* larvae tend to decrease at the end of the last larval instar until pupation. Also, it has been reported that fungal infection can lead to a decrease in the number of hemocytes (Boguś et al., 2018), which can suggest that cellular innate immune response is responsible for controlling *Malassezia* spp. Owing to the fact that once the percentage of hemocytes decreases as part of the physiology of the insect or as part of a virulence factor of the two species of *Malassezia*, the progression of infection retakes, agreeing with previous findings in which *M. pachydermatis* infection could only be established in *Toll*-deficient *Drosophila melanogaster* flies and immunosuppressed Swiss mice (Merkel et al., 2018; Schlemmer et al., 2018).

In contrast to other studies related to fungal infection assays in *G. mellonella* (Achterman et al., 2011; Mesa-Arango et al., 2013; Eisenman et al., 2014; Merkel et al., 2018; Sheehan and Kavanagh, 2019), the highest inoculum concentration (1.5 × 10^9^ CFU/mL) was needed to successfully establish the *M. furfur* and *M. pachydermatis* systemic infection. This agrees with a previous report where nematodes challenged with *M. pachydermatis* showed higher survival than those infected with *C. albicans* (Brilhante et al., 2018). The aforementioned results may be due to the immune response and the low virulence of these yeasts that usually are found in skin mycobiota as commensal microorganisms (Yamasaki et al., 2009; Grice and Dawson, 2017; Schlemmer et al., 2018). The high load inoculum needed and the fact that the infection progress is improved as the number of hemocytes decreases can be an approach to better understand the reason why these two species of *Malassezia* can cause systemic infections in low weight newborns and immunosuppressed patients with a way of entry (intravascular catheter) and nutritional supply (parenteral nutrition with lipid infusion) for *Malassezia*.

The progress of the infection was evaluated through the melanization score, which depended on time and inoculum concentration; moreover, dissemination of the infection was evidenced by the observation of half sectioned larvae under a stereoscope, where a higher presence of nodules close to the microinjection site was found. Also, the number of nodules appeared to increase as the concentration of the inoculum was augmented, agreeing with the previous report on fungal pathogens (Perdoni et al., 2014; Sheehan and Kavanagh, 2019). Furthermore, as previously demonstrated, fungal burden decreased over time, which may be related to the immune response (Merkel et al., 2018; Schlemmer et al., 2018), but it did not show a significant difference between the two species at 33 and 37°C.

A previous study showed that the hydrophobicity of the cell wall of *M. pachydermatis* was higher than the cell wall of *M. furfur*, and also the hydrophobicity of *M. pachydermatis* was the same at 25 and 37°C, while the hydrophobicity of *M. furfur* was stronger at 25°C than at 37°C (Shibata et al., 2009). Hydrophobicity has been related to adherence and biofilm formation, the latter may provide protection from the host immune system (Cannizzo et al., 2007; Buommino et al., 2016; Angiolella et al., 2018), as has been shown before in *C. albicans* (Katragkou et al., 2010; Xie et al., 2012). This may explain why *M. pachydermatis* isolation was higher than *M. furfur* isolation; as matter of fact, the *Malassezia pachydermatis* isolation was 12-folds higher at 37°C than the *M. furfur* isolation, leading to the belief that *M. pachydermatis* can better avoid the immune system than *M. furfur*, and this is more evident at 37°C.

With respect to the percentage of hemocyte populations, the analysis of the interaction between the hemocyte class and the inoculum concentration showed an effect on the percentage of hemocyte populations. It was observed that the percentage of plasmocytes tends to increase with respect to the other four hemocyte classes. This may be due to the fact that these cells are responsible for adherence and phagocytosis, which is the main antifungal immune response, and also that these cells participated in nodule formation (Arteaga Blanco et al., 2017; Boguś et al., 2018; Pereira et al., 2018).

The histological findings revealed the presence of nodules surrounded by hemocytes with melanin deposition as part of the immune response of *G. mellonella*. The location of these nodules was mainly close to the cuticle in association with fat bodies; besides, tissue integrity was disrupted in larvae infected with the highest concentrations, similar to previous findings in other fungal infection assays (Eisenman et al., 2014; Perdoni et al., 2014; Wuensch et al., 2018; Sheehan and Kavanagh, 2019). The presence of yeast of *M. furfur* and *M. pachydermatis* inside the nodules was observed using calcofluor white, confirming infection by these two agents.

In conclusion, it has been demonstrated in the present study that *G. mellonella* is a suitable infection model for *M. furfur* and *M. pachydermatis*. The larvae have shown to successfully respond and control *Malassezia* infection through the innate immune response. However, in this study, the reference strains used were originally isolated from skin lesions (Guého-Kellermann et al., 2010), which may be a bias. Further studies must be done to evaluate different strains from symptomatic and asymptomatic hosts, the systemic infection in immunosuppressed larvae, virulence factor expression and antifungal resistance, allowing mycologists to unravel the host-pathogen interaction that leads *Malassezia* spp. to stay as a commensal microorganism in the skin of healthy patients or to become an opportunistic pathogen in immunosuppressed ones.

## Data Availability Statement

The datasets generated for this study are available on request to the corresponding author.

## Author Contributions

AC, MT, CP, and EP contributed to the design of the work. AC, MT, EP, FR, and HM performed the experiments. AC, MT, and FR were involved in the analysis and interpretation of data. MT wrote the manuscript. AC and CP made revisions. All authors approved the version to be published and agreed to be accountable for all aspects of the work.

## Conflict of Interest

The authors declare that the research was conducted in the absence of any commercial or financial relationships that could be construed as a potential conflict of interest.
